# Grey matter network markers identify individuals with prodromal Alzheimer’s disease who will show rapid clinical decline

**DOI:** 10.1093/braincomms/fcac026

**Published:** 2022-02-08

**Authors:** Wiesje Pelkmans, Ellen M. Vromen, Ellen Dicks, Philip Scheltens, Charlotte E. Teunissen, Frederik Barkhof, Wiesje M. van der Flier, Betty M. Tijms, for the Alzheimer’s Disease Neuroimaging Initiative

**Affiliations:** 1 Alzheimer Center Amsterdam, Department of Neurology, Amsterdam Neuroscience, Vrije Universiteit Amsterdam, Amsterdam UMC, Amsterdam, The Netherlands; 2 Department of Neurology, Mayo Clinic, Rochester, MN, USA; 3 Neurochemistry Laboratory, Department of Clinical Chemistry, Amsterdam Neuroscience, Vrije Universiteit Amsterdam, Amsterdam UMC, Amsterdam, The Netherlands; 4 Department of Radiology & Nuclear Medicine, Amsterdam Neuroscience, Vrije Universiteit Amsterdam, Amsterdam UMC, Amsterdam, The Netherlands; 5 Queen Square Institute of Neurology and Centre for Medical Image Computing, UCL, London, UK; 6 Department of Epidemiology & Biostatistics, Amsterdam Neuroscience, Vrije Universiteit Amsterdam, Amsterdam UMC, Amsterdam, The Netherlands

**Keywords:** Alzheimer’s disease, clinical progression, graph theory, grey matter networks, mild cognitive impairment

## Abstract

Individuals with prodromal Alzheimer’s disease show considerable variability in rates of cognitive decline, which hampers the ability to detect potential treatment effects in clinical trials. Prognostic markers to select those individuals who will decline rapidly within a trial time frame are needed. Brain network measures based on grey matter covariance patterns have been associated with future cognitive decline in Alzheimer’s disease. In this longitudinal cohort study, we investigated whether cut-offs for grey matter networks could be derived to detect fast disease progression at an individual level. We further tested whether detection was improved by adding other biomarkers known to be associated with future cognitive decline [i.e. CSF tau phosphorylated at threonine 181 (p-tau181) levels and hippocampal volume]. We selected individuals with mild cognitive impairment and abnormal CSF amyloid β_1–42_ levels from the Amsterdam Dementia Cohort and the Alzheimer’s Disease Neuroimaging Initiative, when they had available baseline structural MRI and clinical follow-up. The outcome was progression to dementia within 2 years. We determined prognostic cut-offs for grey matter network properties (gamma, lambda and small-world coefficient) using time-dependent receiver operating characteristic analysis in the Amsterdam Dementia Cohort. We tested the generalization of cut-offs in the Alzheimer’s Disease Neuroimaging Initiative, using logistic regression analysis and classification statistics. We further tested whether combining these with CSF p-tau181 and hippocampal volume improved the detection of fast decliners. We observed that within 2 years, 24.6% (Amsterdam Dementia Cohort, *n* = 244) and 34.0% (Alzheimer’s Disease Neuroimaging Initiative, *n* = 247) of prodromal Alzheimer’s disease patients progressed to dementia. Using the grey matter network cut-offs for progression, we could detect fast progressors with 65% accuracy in the Alzheimer’s Disease Neuroimaging Initiative. Combining grey matter network measures with CSF p-tau and hippocampal volume resulted in the best model fit for classification of rapid decliners, increasing detecting accuracy to 72%. These data suggest that single-subject grey matter connectivity networks indicative of a more random network organization can contribute to identifying prodromal Alzheimer’s disease individuals who will show rapid disease progression. Moreover, we found that combined with p-tau and hippocampal volume this resulted in the highest accuracy. This could facilitate clinical trials by increasing chances to detect effects on clinical outcome measures.

## Teaser

Pelkmans *et al*. report that cut-offs for grey matter network properties can detect fast progressing prodromal Alzheimer’s disease individuals with 65% accuracy. When combined with hippocampal volume and phosphorylated-tau markers accuracy increased to 72%. This could facilitate clinical trials by increasing chances to detect treatment effects on clinical outcome measures.

## Graphical Abstract

**Graphical Abstract:**
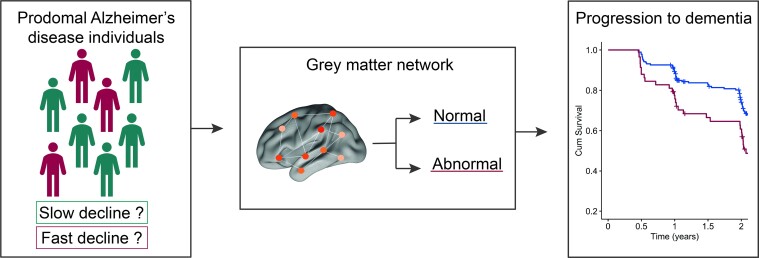

## Introduction

Alzheimer’s disease starts with the aggregation of amyloid-β (Aβ) in the brain, after which it can take up to 20 years for an individual to develop dementia.^1,2^ It has been proposed that Alzheimer's disease clinical trials are most likely to be effective when individuals have biomarker evidence for the presence of Aβ pathology and do not yet show large-scale irreversible neuronal damage.^3,4^ This makes Aβ positive individuals with mild cognitive impairment (MCI), i.e. prodromal Alzheimer's disease, a well-suited population for disease-modifying therapies in Alzheimer's disease clinical trials. However, a challenge faced in secondary prevention trials is that individuals with prodromal Alzheimer's disease show substantial heterogeneity in clinical progression rates.^5^ This heterogeneity hampers the ability to detect treatment effects on cognitive outcomes within a typical 1–2-year clinical trial.^6^ Biomarkers are needed that can help to distinguish individuals with prodromal Alzheimer's disease who will show rapid disease progression from those who will remain stable within a trial time frame.

Previous work has found that disrupted brain grey matter (GM) network measures, reflecting covariance patterns in GM morphology, are related to increased risk of cognitive decline and progression to Alzheimer’s disease dementia.^7–10^ Across those studies, disrupted whole-brain network measures gamma (i.e. normalized values of the clustering coefficient) and small-world, i.e. indicative of an increasingly random network and reduction in small-world organization, were most robustly associated with cognitive decline, adding information to hippocampal volume (HV) and/or CSF tau measures. However, it remains unknown to what extent GM networks can be used to identify single individuals who will show fast progression.

Here we studied this question in individuals with prodromal Alzheimer’s disease from two independent cohorts, i.e. we first established cut-offs in the Amsterdam Dementia Cohort (ADC) and then tested whether these GM network cut-offs could predict if prodromal Alzheimer’s disease subjects remained stable or progressed to dementia within 2 years in the Alzheimer’s Disease Neuroimaging Initiative (ADNI). We then compared the performance of the GM network markers with two other biomarkers known to be associated with the decline in prodromal Alzheimer’s disease [i.e. HV and CSF phosphorylated-tau (p-tau) levels],^11,12^ and determined an optimal model for detecting fast progressors. Finally, we calculated if stratification of prodromal Alzheimer’s disease subjects by abnormal GM network markers would reduce sample size requirements in a hypothetical randomized control trial.

## Materials and methods

### Participants

We studied two cohorts: the ADC and ADNI. The ADC is a memory clinic-based cohort where participants are re-evaluated on a 6-month basis as part of regular care.^13^ The patients in the present study visited the memory clinic between November 2003 and July 2019. ADNI is an ongoing longitudinal research cohort, for which criteria are described in more detail at http://adni.loni.usc.edu/. It was launched in 2003 as a public–private partnership, led by Principal Investigator Michael W. Weiner, MD. The primary goal of ADNI has been to test whether serial MRI, PET, other biological markers and clinical and neuropsychological assessment can be combined to measure the progression of MCI and early Alzheimer’s disease. For up-to-date information, see www.adni-info.org. The data used in the present study were collected between December 2005 and April 2016. The diagnosis was evaluated at 3–12-month intervals. For both cohorts, we selected individuals who fulfilled the consensus criteria for MCI as described by Refs.^14,15^, had abnormal levels of CSF Aβ_(1–42)_, an available baseline structural MRI scan, and at least one follow-up neuropsychological assessment. In ADNI, conversion from MCI to Alzheimer’s disease is reviewed by a central review committee that applies the NINCDS-ADRDA diagnostic criteria^16^ for diagnosis of Alzheimer’s disease dementia. In the ADC, Alzheimer’s disease dementia is also defined according to the NINCDS-ADRDA diagnostic criteria^16^ and from 2011 on the NIA-AA criteria were applied.^17,18^ Disease-modifying trials recruiting prodromal Alzheimer’s disease individuals typically have a trial duration of 24 months or less (Supplementary Table 1),^19^ therefore, we defined individuals as fast progressors when they progressed to dementia within 2 years. In both ADNI and ADC, all participants gave written informed consent for participation in the study and for reuse of the data. Ethical approval was given by the regional ethics committees.

### MRI acquisition and preprocessing

In ADC, structural T_1_-weighted images were acquired on nine different scanners, using a standardized protocol as part of routine patient care, of which the acquisition parameters are described in detail in the Supplementary material. In ADNI, T_1_-weighted scans were performed on 1.5 or 3 T scanners using previously described standardized protocols,^20^ typically a sagittal 3D MP-RAGE with a voxel size of 1.2 mm^3^. All images were segmented into GM, white matter and CSF using the Statistical Parametric Mapping (SPM12, https://www.fil.ion.ucl.ac.uk/spm/software/spm12/) running in MATLAB (v2011a). The segmented GM images were resliced to 2 × 2 × 2 mm isotropic voxels to reduce the dimensionality of the data. Total intracranial volume (TIV) was computed as the sum of GM, white matter and CSF volumes. The automated anatomical labelling atlas was used to obtain hippocampal GM volume estimates.^21^ A previously determined cut-off was applied to determine hippocampal abnormality in ADNI with a mean HV corrected for TIV of >3.68 ml.^22^ All GM segmentations were visually checked for quality.

### Single-subject GM networks

Single-subject GM networks were constructed from the native GM images as described in the freely available MATLAB scripts: https://github.com/bettytijms/Single_Subject_Grey_Matter_Networks and in more detail in Tijms *et al.*^23^ For each individual, a network was determined from the native space GM segmentations. First, nodes were defined as cubes of 3 × 3 × 3 voxels (6 mm × 6 mm × 6 mm) using an atlas-free approach. The nodes keep the 3D structure of the cortex intact, and thereby contain information on GM intensity as well as spatial information between the voxels. Next, connections were defined when nodes showed structural similarity as determined with the Pearson correlation coefficients across corresponding voxels. In order to find the maximum correlation value with a target cube across the curved cortex, each cube was rotated by an angle with multiples of 45° over all axes. The resulting similarity matrix containing all pairwise correlations was binarized using a threshold that reduced the chance of spurious correlations in the network to 5%. This corresponds to a significance level of *P* < 0.05 corrected for multiple comparisons using a permutation-based procedure.^24^

For each individual GM network, we calculated normalized clustering coefficient (*γ*), normalized path length (*λ*) and the small-world coefficient (*σ*), as our previous studies showed that these measures are most robustly associated with cognitive decline.^7–9^ Briefly, *γ* quantifies how a network’s clustering coefficient (the fraction of a node’s neighbours that are also neighbours of each other) deviates from a random network. *λ* quantifies how a network’s path length (the shortest path length between all pairs of nodes in the network) deviates from a random network. In more detail, we divided the average clustering coefficient and path length values by those values of five randomized reference networks of identical size and degree distribution.^25^ The ratio of *γ* to *λ*, is defined as the *small-world coefficient*, indicative of the optimal balance between information segregation and integration. The network measures were computed with scripts from the Brain Connectivity Toolbox (https://sites.google.com/site/bctnet/),^26^ modified for large-scale networks.

### CSF analysis

Lumbar puncture was performed as described in Mulder *et al.*^27^ and Engelborghs *et al.*^28^ for ADC, and for ADNI according to the ADNI procedures manual (http://www.adni-info.org/). CSF concentrations of Aβ_(1–42)_ and tau phosphorylated threonine 181 were measured using sandwich ELISAs (Innotest, Innogenetics, Belgium), at the Neurochemistry laboratory of the Department of Clinical Chemistry of the Amsterdam University Medical Center (ADC), and for ADNI with the multiplex xMAP Luminex platform (Luminex Corp, Austin, TX, USA) and INNO-BIA AlzBio3 (Innogenetics, Ghent, Belgium) immunoassay kit-based reagents. The cut-offs for CSF Aβ_(1–42)_ and p-tau abnormality have previously been determined and were 813 and 52 pg/ml for ADC,^27,29^ and 192 and 23 pg/ml for ADNI.^30^ Because CSF p-tau was used as one of the predictors for decline, we only used an amyloid marker to define prodromal Alzheimer’s disease.

### Statistical analysis

Prognostic cut-offs for GM network measures (*γ*, *λ* and *σ*) to predict progression to dementia within 2 years were determined in the ADC cohort through time-dependent receiver operating characteristic (tROC) analysis from censored survival data using nearest neighbour estimation.^31^ The advantage of tROC analyses over standard ROC is that tROC takes the time to an event into account when calculating the sensitivity, specificity and area under the curve (AUC) for a specific marker. For each network measure, we determined the optimal cut-off value in the ADC that best separated prodromal Alzheimer’s disease patients with a high or low risk for fast clinical decline at 2 years post-baseline. We then used these cut-offs in ADNI to evaluate detecting of fast progressors using logistic regression analysis, and reported accuracy, sensitivity and specificity. We further evaluated whether GM network measures provide additive information to more commonly used biomarkers for Alzheimer’s disease, CSF p-tau and HV, by adding the latter markers to the logistic regression model and compared model fit using the Akaike’s Information Criterion (AIC). Analyses were initially performed without covariates as such a model would be easiest to apply in practice. We repeated the analyses adding sex, age, education and MRI scanner as covariates. Next, we tested the extent to which one, two or three abnormal biomarkers (small-world, p-tau, HV) could predict who progressed to dementia within 2 years using logistic regression. Finally, sample sizes were estimated for a hypothetical 2-year randomized-controlled trial with two arms, showing an expected treatment effect of 25% reduction of decline on the Mini-Mental State Examination (MMSE)^32^ and the Clinical Dementia Rating scale—Sum of Boxes (CDR-SB),^33^ when stratifying for GM network abnormality using the following formula:

$$\mathrm{Sample}\;\mathrm{size}\;/\;\mathrm{arm}=2(z_{1-\alpha /2}+z_{1-\beta }{)}^{2}\;x\;(\sigma_{b}^{2}+\sigma_{e}^{2}\;/\sum (t_{i}\text{–}t_{\mathrm{mean}}{)}^{2})\;/\Delta^{2}$$

where *α* is equal to the Type I error of a two-sided significance test set at 0.05, and the power (1 − *β*) is 80%. From the linear mixed model, σ_b_^2^ and σ_e_^2^ are the variance in random subject slopes and the residual error variance, respectively. *t_i_* is the measurement at time *i* and *t*_mean_ is the average follow-up time, Δ is the difference in mean rate of decline in the treatment versus control group using a 25% treatment effect. Analyses were conducted with R version 4.1.1. using the survival and survivalROC packages.^31^

### Data availability

The data that support the findings of this study can be made available on request (ADC) or are publicly available (ADNI).

## Results

### Study population

A total of 491 prodromal Alzheimer’s disease patients were available for this study (Table 1). The participants from ADNI were older and had on average more years of education compared with ADC participants. After 2 years, 60 (24.6%) subjects in ADC and 84 (34.0%) subjects in ADNI showed clinical progression to dementia.

**Table 1:** Subject characteristics

	ADC	ADNI
*n*	244	247
Age (y)	67.5 (7.4)	72.9 (7.0)
Sex (f)	113 (46.3%)	104 (42.1%)
Education (y)	11.7 (3.2)	15.8 (2.8)
MMSE	26.4 (2.4)	27.5 (1.8)
Progression to dementia (2 y)	60 (24.6%)	84 (34.0%)
*APOEe4* carrier	157 (73.0%)	164 (66.4%)

Data are presented as mean (SD) or *n* (%); ADC, Amsterdam Dementia Cohort; ADNI, Alzheimer’s Disease Neuroimaging Initiative; y, years; f, female; MMSE, Mini-Mental State Examination; APOE, Apolipoprotein E.

### Cut-points for GM network measures

**Figure 1:**
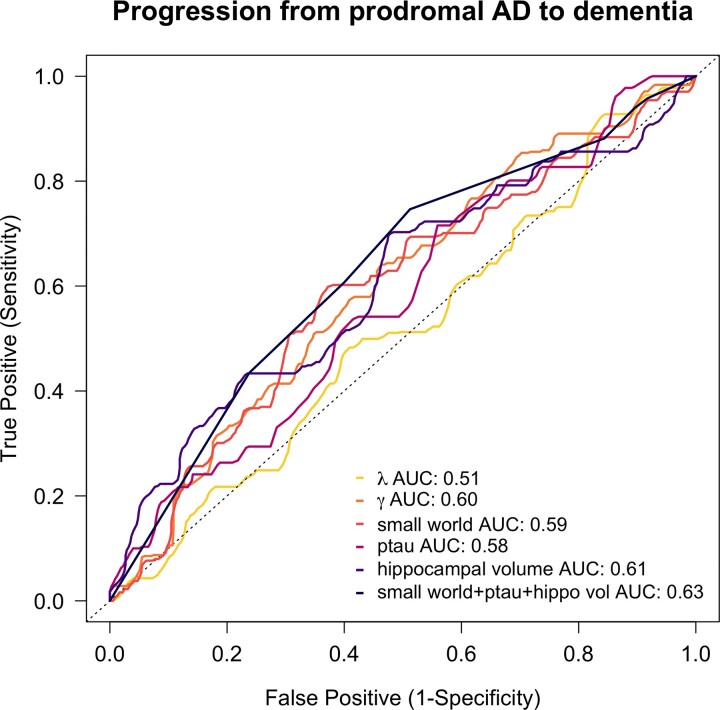
**tROC analyses of prognostic biomarkers for predicting clinical progression within 2 years in ADC**. tROC curves and corresponding areas under the curves to determine the most optimal cut-off for GM network markers together and CSF p-tau and normalized HV to assess accuracy when predicting clinical progression to dementia within 2 years post-baseline in prodromal Alzheimer’s disease individuals (*n* = 244).

First, we determined cut-points in ADC optimizing classification of prodromal Alzheimer’s disease individuals who remained stable versus those who progressed to dementia within 2 years. Applying the tROC analysis yielded the following cut-offs: 1.627 for *γ*, 1.106 for *λ* and 1.479 for *σ*. These cut-offs resulted in AUCs of 0.60 for *γ* (sensitivity = 64%, specificity = 54%), 0.51 for *λ* (sensitivity = 93%, specificity = 16%) and 0.59 for *σ* (sensitivity = 60%, specificity = 63%; Fig. 1). This was comparable to the AUCs of 0.58 for p-tau (sensitivity = 72%, specificity = 44%) and 0.61 for HV (sensitivity = 70%, specificity = 52%).

### Predicting fast clinical progression using GM network measures

**Figure 2:**
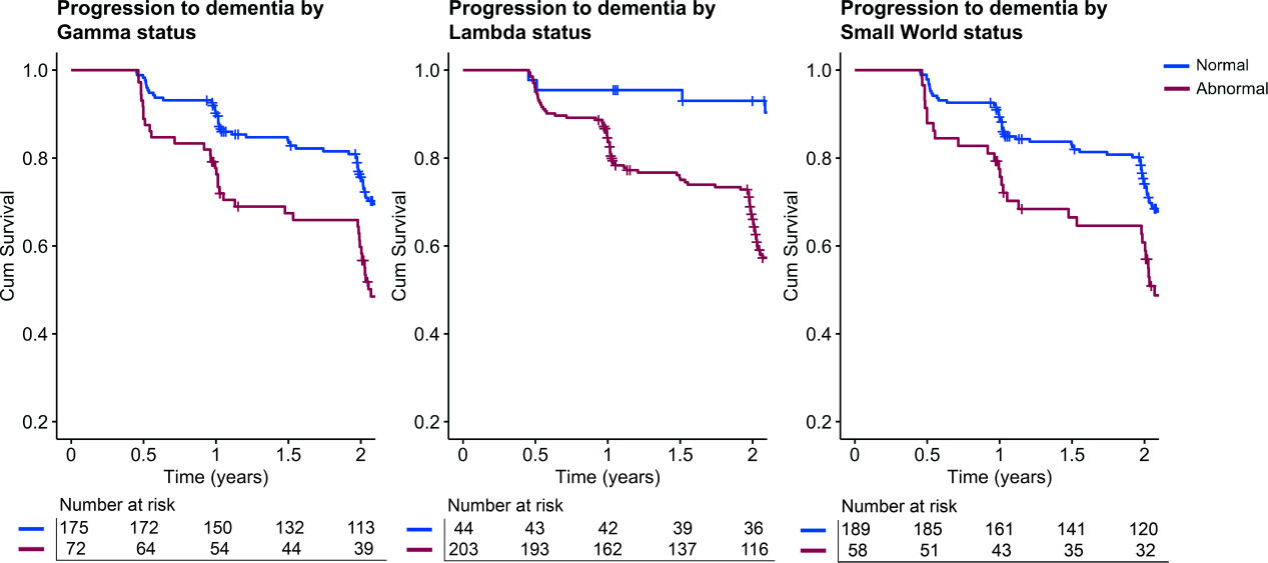
**Kaplan–Meier curves of progression from prodromal Alzheimer’s disease to dementia within 2 years in ADNI**. Lines represent individuals with normal (blue) and abnormal (red) GM network values. GM network cut-offs were determined in ADC and applied in ADNI.

We next performed logistic regression analysis in ADNI to evaluate detection of fast progression using ADC determined cut-points. GM network measures (*γ* and *σ*) showed an accuracy of 65% (sensitivity = 33–42%, specificity = 77–82%) for predicting stable versus progressing individuals. Compared with individuals with normal *γ* values, those with abnormal *γ* values were 2.4 times [95% confidence interval (CI) = 1.4–4.3] more likely to progress to dementia within a 2-year period (Table 2 and Fig. 2). Similar odds ratios (ORs) were observed for individuals with abnormal small-world values [OR = 2.2 (95% CI = 1.2–4.1)], and abnormal HV [OR = 2.9 (95% CI = 1.6–5.2); Table 2 and Fig. 2]. Individuals with abnormal *λ* and CSF p-tau values showed a higher risk of progression to dementia, that is an OR of 6.5 (95% CI = 2.2–18.9) and an OR of 3.1 (95% CI = 1.0–9.4), respectively, however, this was accompanied with larger CIs and low accuracy values <0.5. When correcting for sex, age, education and MRI scanner, the ORs for abnormal biomarkers to predict progression increased (Supplementary Table 2). In addition, the mixed model analysis showed that individuals with abnormal GM network values showed steeper decline on the MMSE (*β* ± SE, *γ*: −1.00 ± 0.25; *λ*: −0.78 ± 0.30; *σ*: −0.92 ± 0.26), and faster deterioration as measured by the CDR-SB (*β* ± SE, *γ*: 0.60 ± 0.15; *λ*: 0.53 ± 0.19; *σ*: 0.62 ± 0.16), compared with individuals with normal GM network metrics (Supplementary Fig. 1).

**Table 2:** Odds ratios of abnormal biomarkers to predict clinical progression in ADNI

	OR (CI)	Se	Sp	Acc	*P*-value
Gamma	2.43 (1.38–4.29)	0.42	0.77	0.65	0.002*
Lambda	6.50 (2.24–18.88)	0.95	0.25	0.49	<0.001*
Small-world coefficient	2.22 (1.21–4.05)	0.33	0.82	0.65	0.010*
P-tau	3.12 (1.04–9.38)	0.95	0.13	0.41	0.043*
Hippocampal volume	2.90 (1.61–5.20)	0.40	0.81	0.67	<0.001*

ORs of logistic regression analysis for progression of prodromal Alzheimer’s disease subjects to dementia within 2 years. GM network cut-offs were determined in ADC and applied to ADNI. Results are shown for every abnormal biomarker with 95% CIs. CI, confidence interval; Se, sensitivity; Sp, specificity; Acc, accuracy.

* *P* < 0.05.

### Optimal biomarker model to identify fast progression

Next, we assessed in ADNI whether GM network measures contained information on disease progression complementary to p-tau and HV. For these analyses, we assessed the small-world coefficient only, as it can be considered a summary measure of both normalized clustering and normalized path length. A model including only p-tau resulted in an AUC of 0.54 (AIC = 316), adding HV improved the model fit (AUC = 0.64; AIC = 304, *P* < 0.001). When adding the small-world coefficient, the model did not improve significantly (AUC = 0.67; AIC = 303, *P* = 0.082) for the classification of rapid decliners (Supplementary Table 3). Note that the CSF p-tau and HV cut-offs were previously established using ADNI-specific cut-points,^22,30^ while the small-world cut-off was based on the independent ADC, which suggests that the p-tau and HV might provide over-optimistic model performance. When the small-world cut-off was also based on ADNI, the model did improve significantly (AUC = 0.70; AIC = 291, *P* < 0.001; Supplementary Table 3).

**Figure 3:**
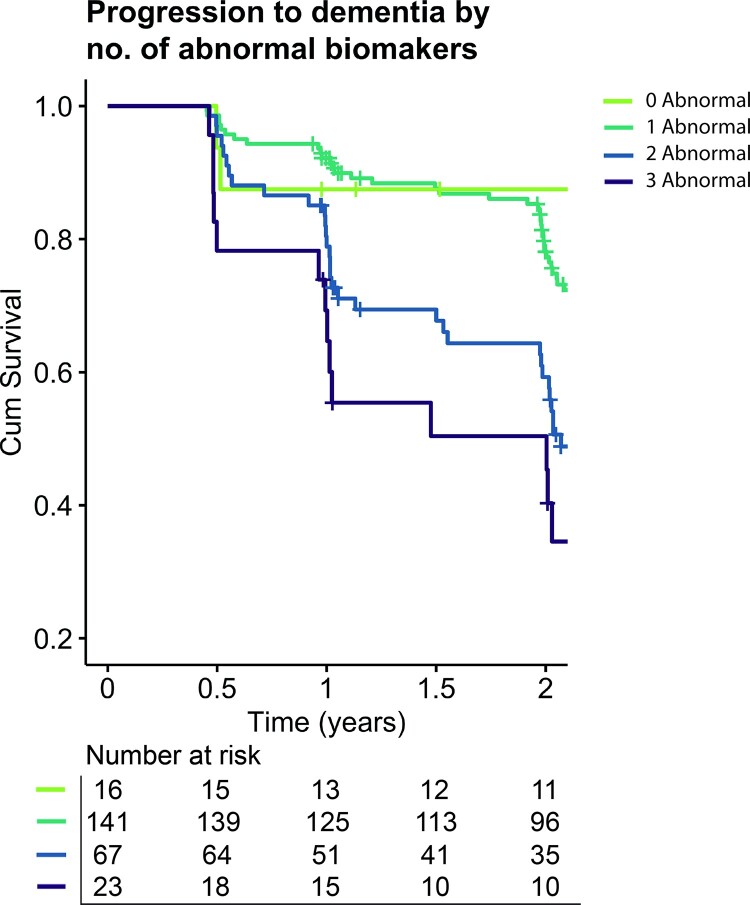
**Kaplan–Meier curves of progression from prodromal Alzheimer’s disease to dementia within 2 years in ADNI**. Separate lines represent individuals with zero, one, two or three abnormal biomarkers (GM network small-world topology, cerebrospinal fluid p-tau and hippocampal volume).

We then tested whether combining the biomarkers would improve detection of fast progressors, by labelling individuals as having no, one, two or three abnormal predictors. A gradual increase in rapid progression risk was observed with the number of abnormal biomarkers (Table 3 and Fig. 3). Showing a 6.4 times increased risk of rapid clinical decline with two abnormal prognostic biomarkers when compared with individuals with abnormal Aβ only. This risk increased steeply to an OR of 10.9 for three abnormal prognostic biomarkers (Table 3 and Fig. 3). Rapid progression could be most accurately identified for individuals with all three biomarkers abnormal, with an accuracy of 72% (sensitivity = 88%, specificity = 61%).

**Table 3:** Combining prognostic biomarkers for predicting rapid progression to dementia

	OR (CI)	Se	Sp	Acc	*P*-value
One abnormal biomarker	2.40 (0.52–11.07)	0.95	0.12	32%	0.262
Two abnormal biomarkers	6.40 (1.35–30.37)	0.94	0.29	55%	0.019*
Three abnormal biomarkers	10.89 (1.99–59.72)	0.88	0.61	72%	0.006*

ORs of logistic regression analysis in ADNI for the combination of abnormal biomarker predictors. Biomarker combination contains abnormal small-world coefficient, p-tau and hippocampal volume; reference category is all normal biomarkers; Se, sensitivity; Sp, specificity; Acc, accuracy.

* *P* < 0.05.

### Sample size estimates

We next studied if sample size estimates for clinical trials to detect a 25% slowing in the rate of decline on the MMSE and CDR-SB would reduce when adding network measures. Table 4 shows for the prodromal Alzheimer’s disease cohort (Aβ^+^ column) without additional markers an estimated sample size of 729 (95% CI = 444–1364) for the MMSE, and 486 (95% CI = 348–737) for the CDR-SB. Estimated sample sizes were smallest when restricting enrolment to prodromal Alzheimer’s disease participants with abnormal p-tau, abnormal HV and abnormal small-world status (Aβ^+^*σ*^+^ p-tau^+^ HV^+^, Table 4).

**Table 4:** Sample size estimates for a hypothetical 2-year trial in prodromal Alzheimer’s disease subjects by biomarker abnormality

	MMSE	CDR-SB
Aβ^+^	729 [444–1364]	486 [348–737]
Aβ^+^ σ^+^	493 [231–1530]	370 [214–833]
Aβ^+^ p-tau^+^	717 [430–1377]	445 [318–676]
Aβ^+^ HV^+^	385 [195–965]	310 [192–609]
Aβ^+^ σ^+^ p-tau^+^	467 [218–1471]	347 [201–779]
Aβ^+^ σ^+^ HV^+^	398 [151–2162]	263 [133–820]
Aβ^+^ p-tau^+^ HV^+^	392 [194–1047]	284 [174–569]
Aβ^+^ σ^+^ p-tau^+^ HV^+^	358 [138–1864]	262 [131–853]

Sample size estimates and 95% CIs for a hypothetical 2-year randomized-controlled trial with two arms required to detect a 25% reduction of decline in cognitive outcome measures with a power of 80% using ADNI data. Aβ^+^, MCI individuals who have abnormal CSF amyloid β_1–42_ levels; Aβ^+^ σ^+^, MCI individuals who both have abnormal Aβ and abnormal GM network small-worldness; Aβ^+^ p-tau^+^, MCI individuals who both have abnormal Aβ and abnormal p-tau_181_ CSF levels; Aβ^+^ HV^+^, MCI individuals who both have abnormal Aβ and abnormal HV; etc.; MMSE, Mini-Mental State Examination; CDR-SB, Clinical Dementia Rating scale—Sum of Boxes.

## Discussion

The main finding of the present study is that GM network measures can aid in identifying individuals with prodromal Alzheimer’s disease who are likely to progress to dementia within the next 2 years. Models combining small-world coefficient, p-tau and HV showed the best ability to detect progression. These findings could increase power in Alzheimer’s disease trials by selecting those individuals with abnormal GM network characteristics at high risk for clinical progression within a time frame of 24 months.

Most studies so far that investigated prognostic markers in individuals with MCI, studied also subjects with normal Aβ values.^5,34–40^ Such an approach makes it difficult to distinguish between the effects caused by Aβ, tau and neuronal injury on cognition, as abnormalities in tau and neurodegeneration are closely related to Aβ pathology.^41–43^ Moreover, it can also inflate accuracy statistics, because abnormal Aβ has a strong predictive effect of decline.^44,45^ Predicting progression within Aβ positive individuals is, however, more difficult and the few longitudinal studies that investigated prognostic markers within prodromal Alzheimer’s disease patients have demonstrated a more modest predictive value over Aβ.^12,46^ This is also reflected by the relatively modest AUC values in the present study. Moreover, in contrast to previous studies showing that a more disorganized GM network is associated with cognitive decline,^7,8,47^ our findings indicate that an individual’s GM network measure can be classified as normal or abnormal which is relevant for clinical application and inclusion of subjects in therapeutic trials to select those individuals who will show fast progression over a relatively short time frame.^19^

GM network measures start to change early in the disease process: previous studies indicate that the presence of Aβ significantly alters GM networks, and that these alterations may precede tau and neurodegeneration,^48–51^ and can predict future hippocampal atrophy.^52^ It could be hypothesized that the Alzheimer’s disease neuropathological changes contribute to the observed brain network disruptions, and represent a close biological substrate for disease progression and cognitive decline in Alzheimer’s disease. In the current study, we show that individuals with a more random network, as reflected by an abnormal small-world topology, were more than twice as likely to progress to dementia compared with those with normal values. Still, single biomarkers showed modest accuracy to predict fast progression.^34,53^ In the present study, we found that the highest predictive accuracy was obtained when the small-world coefficient was combined with both p-tau and HV. This resulted in an OR of >10 (sensitivity 88%, specificity 61%, accuracy 72%) for progression to dementia within 2 years for individuals with all three biomarkers abnormal and a 46–60% reduction in required sample size to detect at 25% treatment effect in a hypothetical 2-year trial compared with abnormal amyloid alone. This is in line with previous studies^54–56^ that showed reduced sample size estimates when tau and/or neuronal injury markers are abnormal. We show that GM network measures may further improve predictive models. Together, these studies and our results provide further support for the idea that combining multiple markers may facilitate clinical trials by increasing chances to detect effects on clinical outcome measures. Strengths of this study include that GM network cut-offs were determined in one cohort and then showed to be generalizable to an independent cohort. Secondly, our approach allows for patient-level application. However, a follow-up study is likely needed to further investigate the prognostic value of the determined cut-offs in a larger sample. Another important next step would be to develop more user-friendly software and investigate whether GM network cut-points can be applied to challenges in a clinical setting, such as aiding in short-term care planning for dementia patients.

## Conclusion

In conclusion, we showed that GM network measures can be applied to identify individuals with prodromal Alzheimer’s disease at risk for fast progression. Moreover, when combined with p-tau and HV this resulted in the highest prognostic accuracy, which could contribute to detect treatment effects in Alzheimer’s disease clinical trials.

## Supplementary Material

fcac026_Supplementary_Data

## Abbreviations

Aβ =: amyloid-β
AD =: Alzheimer's disease
ADC =: Amsterdam Dementia Cohort
ADNI =: Alzheimer’s Disease Neuroimaging Initiative
AIC =: Akaike’s Information Criterion
AUC =: area under the curve
CDR-SB =: Clinical Dementia Rating scale—Sum of Boxes
CI =: confidence interval
*γ* =: normalized clustering coefficient
GM =: grey matter
HV =: hippocampal volume
*λ*: normalized path length
MCI =: mild cognitive impairment
MMSE =: Mini-Mental State Examination
OR =: odds ratio
p-tau =: phosphorylated-tau
*σ* =: small-world coefficient
SPM =: Statistical Parametric Mapping
TIV =: total intracranial volume
tROC =: time-dependent receiver operating characteristic
