# Supplementary material for: Grey matter network markers identify individuals with prodromal Alzheimer’s disease who will show rapid clinical decline
Source: Brain Commun. 2022 Feb 8;4(2):fcac026. doi: 10.1093/braincomms/fcac026 (PMC8924646; doi:10.1093/braincomms/fcac026)
Supplement: fcac026_Supplementary_Data [file fcac026_supplementary_data.docx]

**Supplementary Material**

**Supplementary information**

***MRI acquisition parameters in ADC***

﻿Structural T1-weighted images were acquired as part of routine patient care from nine different scanners. The following parameters were used: 1.5T Siemens Avanto: Magnetization prepared rapid acquisition gradient echo (MPRAGE), coronal plane, repetition time (TR) 2700 ms, echo time (TE) 5.2 ms, inversion time (TI) 950 ms, flip angle (FA) 8°, voxel size 1×1×1.5 mm^3^; 3T GE Discovery MR750: ﻿FSPGR, sagittal plane, TR 7.8 ms, TE 3ms, FA 12°, voxel size 1 mm^3^; 1T Siemens Magnetom Impact: MPRAGE, coronal plane, TR 15 ms, TE 7 ms, TI 300 ms, FA 15°, voxel size 1×1×1.5 mm^3^; 3T Philips Ingenuity PET/MR system: sagittal turbo field echo (TFE), sagittal plane, TR 7 ms, TE 3 ms, FA 12°, voxel size 1×1×1 mm^3^; 1.5T GE SignaHDxt: sagittal fast spoiled gradient echo (FSPGR), sagittal plane, TR 12.4 ms, TE 5.17 ms, TI 450 ms, FA 12°, voxel size 0.98×0.98×1.5 mm^3^; 3T GE SignaHDxt: FSPGR, sagittal plane, TR 708 ms, TE 7 ms, FA 12°, voxel size 0.98×0.98×1 mm^3^; 1.5T Siemens Sonata: MPRAGE, coronal plane, TR 2700 ms, TE 3.97 ms, TI 950 ms, FA 8°, voxel size 1×1×1.5 mm^3^; Toshiba Titan 3T: sagittal fast field echo (FFE) sequence (TR = 9, TE = 3, TI = 800, FA = 7°, 1.00 x 1.00 x 1.00 mm voxels); 1.5T Siemens Vision: MPRAGE, coronal plane, TR 15 ms, TE 7 ms, FA 8°, voxel size 0.98×0.98×1.5 mm^3^.

| **Supplementary Table 1** | |  |  |  |
| --- | --- | --- | --- | --- |
| Overview of current phase 2 and phase 3 disease modifying trials in prodromal AD | | | |  |
| *< 1 year* | *1 year* | *1.5 years* | *2 years* | *> 2years* |
| AMX0035 | GV-971 | ABvac40 | AL002 | JNJ-63733657 |
| BPN14770 | Benfotiamine | ACI-35 | Blarcamesine |  |
| L-Serine | Curcumin | Aducanumab | Gantenerumab |  |
| Lamivudine | Dasatinib + Quercetin | AGB101 | Metformin |  |
| Neflamapimod | Deferiprone | ALZ-801 | Tilavonemab |  |
| Nicotinamide | Levetiracetam | Azeliragon | Zagotenemab |  |
| Pepinemab | Liraglutide | Donanemab |  |  |
| Posiphen | Montelukast | Edonerpic (T-817) |  |  |
| PU-AD | Rapamycin | Gosuranemab (BIIB092) |  |  |
| Tacrolimus |  | IVIg |  |  |
| TEP |  | Lecanemab |  |  |
|  |  | Lenalidomide |  |  |
|  |  | Omega 3 PUFA |  |  |
|  |  | PQ912 |  |  |
|  |  | Semorinemab |  |  |
|  |  | Simufilam |  |  |
|  |  | Solanezumab |  |  |
|  |  | Valacyclovir |  |  |
| Source: https://www.clinicaltrials.gov | |  |  |  |

| **Supplementary Table 2.** | |  |  |  |
| --- | --- | --- | --- | --- |
| Odds ratios of abnormal biomarkers to predict clinical progression in ADNI | | |  |  |
|  | Odds (CI) | p-value |  |  |
| Gamma | 3.35 (1.65 - 6.80) | 0.001* |  |  |
| Lambda | 7.05 (2.27 - 21.92) | 0.001* |  |  |
| Small-world coefficient | 2.99 (1.43 - 6.25) | 0.004* |  |  |
| P-tau | 3.86 (1.06 - 14.07) | 0.040* |  |  |
| Hippocampal volume | 2.46 (1.22 - 4.95) | 0.012* |  |  |
| Odds ratios of logistic regression analysis for progression of prodromal AD subjects to dementia within two years. GM network cut-offs were determined in ADC and applied to ADNI. Results are shown for every abnormal biomarker with 95% confidence intervals; Model is adjusted for age, sex, education, and MRI scanner; CI: confidence interval; * *p*<0.05. | | |  |  |
|  |  |  |  |  |
|  |  |  |  |  |
|  |  |  |  |  |

| **Supplementary Table 3.** |  |  |  |  |
| --- | --- | --- | --- | --- |
| Model fit comparisons for predicting rapid progression to dementia. | | | |  |
|  | AUC (CI) | AIC | *p*-value |  |
| Model 1: p-tau | 0.54 (0.51 - 0.58) | 315.6 |  |  |
| Model 2: p-tau + HV | 0.64 (0.57 - 0.70) | 304.0 | <0.001* |  |
| Model 3a: p-tau + HV + small-world | 0.67 (0.60 - 0.73) | 303.0 | 0.082 |  |
| Model 3b: p-tau + HV + small-world | 0.70 (0.64 - 0.77) | 291.4 | <0.001* |  |
| Output of logistic regression analyses in ADNI. The AIC represents the model fit. The *P*-value for model difference compares a model with a less complex model (that is model 2 vs model 1, and model 3 vs 2). Model3a small-world abnormality cutpoint determined in ADC; Model3b small-world abnormality cutpoint determined in ADNI. Abbreviations: AUC = Area under Curve; CI = Confidence Interval; AIC = Akaike’s Information Criterion; HV = Hippocampal Volume. | | | |  |
|  |  |  |  |  |
|  |  |  |  |  |
|  |  |  |  |  |
|  |  |  |  |  |

**Supplementary Figure 1**

**
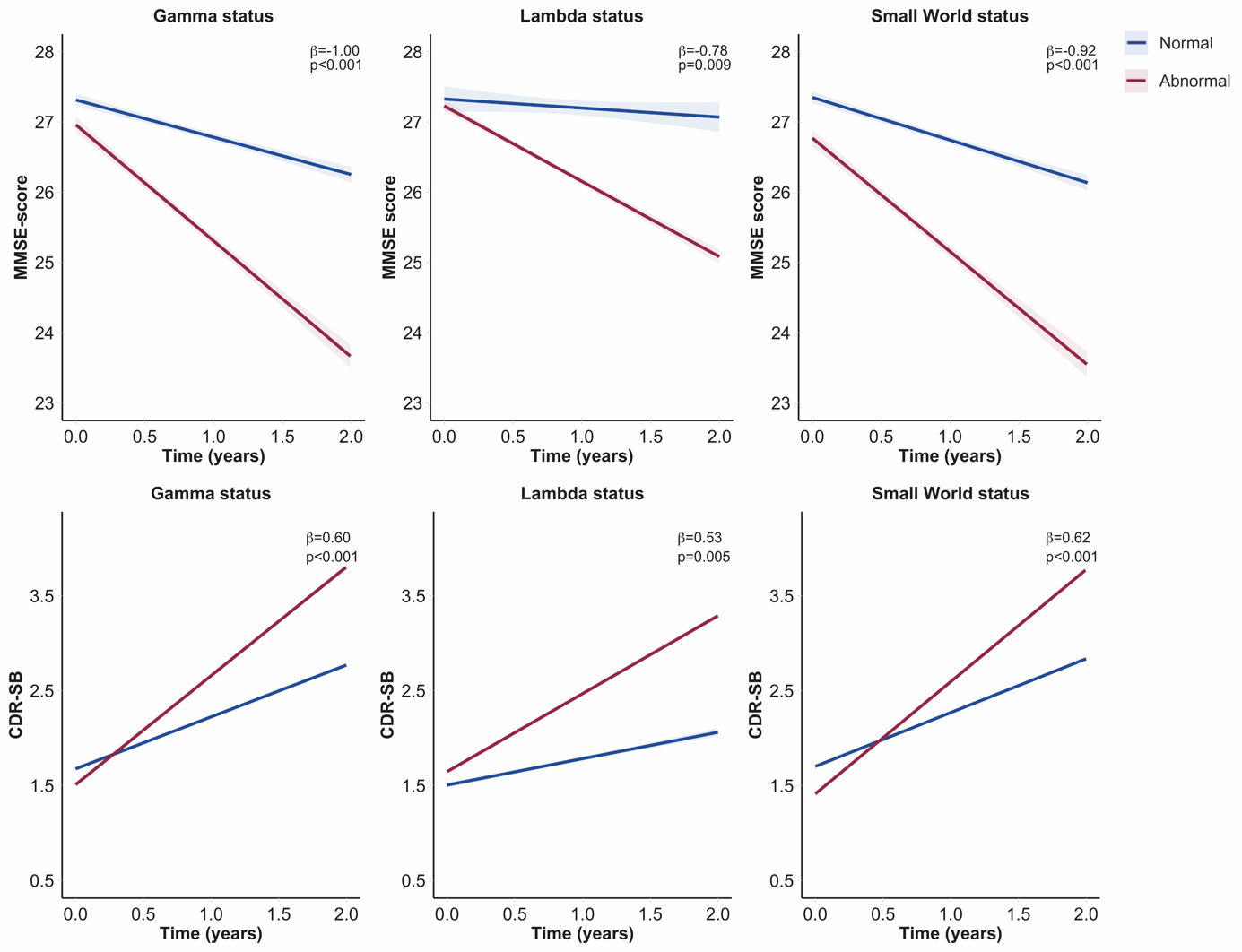
**

Cognitive performance over time by grey matter network status. Linear mixed model analysis with a random intercept and slope. Standardised beta estimates are adjusted for age, sex, education, and scanner type.
